# Functional independence trajectories over 5 years in older veterans with traumatic brain injury: A model systems study

**DOI:** 10.1002/pmrj.13312

**Published:** 2025-01-27

**Authors:** Mia E. Dini, Rachel E. Wallace, Daniel W. Klyce, Carmen M. Tyler, Michael Vriesman, Shannon B. Juengst, Victoria Liou‐Johnson, Kelli W. Gary, Kristen Dams‐O'Connor, Raj G. Kumar, Umesh M. Venkatesan, Kritzia Merced, Paul B. Perrin

**Affiliations:** ^1^ Research Service, Central Virginia Veterans Affairs Health Care System Richmond Virginia USA; ^2^ Department of Psychology University of Virginia Charlottesville Virginia USA; ^3^ Mental Health Service Central Virginia Veterans Affairs Health Care System Richmond Virginia USA; ^4^ Department of Physical Medicine and Rehabilitation Virginia Commonwealth University Health System Richmond Virginia USA; ^5^ Inpatient Psychology Service Sheltering Arms Institute Richmond Virginia USA; ^6^ Department of Psychology Virginia Commonwealth University Richmond Virginia USA; ^7^ Brain Injury Research Center The Institute for Rehabilitation Research, Memorial Hermann Houston Texas USA; ^8^ Polytrauma Department VA Palo Alto Healthcare Center Palo Alto California USA; ^9^ Clinical Excellence Research Center Stanford University School of Medicine Stanford California USA; ^10^ Department of Rehabilitation Counseling Virginia Commonwealth University Richmond Virginia USA; ^11^ Department of Rehabilitation and Human Performance and Department of Neurology Icahn School of Medicine at Mount Sinai New York New York USA; ^12^ Department of Rehabilitation and Human Performance Icahn School of Medicine at Mount Sinai New York New York USA; ^13^ Jefferson Moss Rehabilitation Research Institute Elkins Park Pennsylvania USA; ^14^ Department of Rehabilitation Medicine Sidney Kimmel Medical College at Thomas Jefferson University Philadelphia PA USA; ^15^ School of Data Science, University of Virginia Charlottesville Virginia USA

## Abstract

**Background:**

Research on older adults who sustain a traumatic brain injury (TBI) has predominantly been on civilian, nonveteran populations. Military populations experience higher rates of TBI and often experience the additive effects of TBI and other comorbid disorders, including posttraumatic stress disorder and/or substance use that may increase disability over time.

**Objective:**

To investigate predictors of functional independence trajectories over the 5 years after TBI in veterans 55 years or older at injury.

**Setting:**

Five Veterans Affairs (VA) polytrauma rehabilitation center (PRC) inpatient rehabilitation programs.

**Participants:**

Veterans who experienced their TBI at 55 years or older and had completed one or more Functional Independence Measure (FIM) Motor and Cognitive measure at 1, 2, or 5 years after TBI (*n* = 184) from the VA TBI Model Systems national database.

**Design:**

Retrospective analysis of observational data using hierarchical linear models.

**Main Measures:**

FIM Motor and Cognitive scores at 1, 2, and 5 years after TBI.

**Results:**

Motor and cognitive functioning decreased over time. Lower FIM Motor trajectories occurred in participants who had pre‐TBI functional limitations in going out of the home and with longer posttraumatic amnesia (PTA). FIM Motor scores decreased over time, and the decrease was steeper for those with a moderate or severe injury. Lower FIM Cognitive trajectories occurred in participants who had problematic substance use at baseline and among those with longer PTA. FIM Cognitive scores decreased at a steeper rate for participants with greater injury severity.

**Conclusions:**

Similar to previously published studies in civilian populations, older veterans with TBI may be at risk for functional and cognitive decline. This study's findings increase the field's understanding of functional trajectories after TBI in older adults and may help identifty those who are at risk for lower functional outcomes.

Rates of traumatic brain injury (TBI) have increased in recent years, with over 69,000 TBI‐related deaths in the United States in 2021^1^ and 223,135 nonfatal TBI‐related hospitalizations in 2018.^2^ Outcome trajectories are variable, resulting in short‐ and long‐term disability for many survivors. Mortality and morbidity rates are higher for older adults relative to other age cohorts.^3^ Among those hospitalized for a nonfatal TBI, rates of injury are highest in adults ≥75 years old (321.4 per 100,000), and unintentional falls remain the leading mechanism of injury in this group.^2^ Americans are living longer, and nearly one in four Americans will be 65 years or older by 2060.^4^ Thus, rates of older adults aging with TBI will increase, and there will be a continued need to understand longitudinal outcomes of older adults with TBI and related sequelae.

The prognostic value of TBI severity is strongly influenced by the age of the person at the time of injury, with older adults experiencing worse functional outcomes than individuals who were younger at injury.^5^, ^6^, ^7^, ^8^ Broadly, some degree of functional decline may be expected for older adults, with one study finding that 42% of older adults aged 60–70 with no functional deficits at baseline experienced intermediate or severe functional decline over 9 years.^9^ The risk for poor functional outcomes among older adults appears to be exacerbated by comorbid health problems—both pre‐ and postinjury—as well as complications experienced during acute care treatment for new‐onset TBI.^10^, ^11^, ^12^, ^13^ As older adults with TBI continue to age, they also accrue increased risk for poor functional outcomes, greater overall disability, and reduced community participation.^6^, ^14^, ^15^ Individuals tend to experience functional improvements early in the course of rehabilitation after TBI with functional decline in later years.^16^ As individuals age with TBI, both motor and cognitive functional declines have been linked to endocrine, cardiovascular, oncologic, and neuropsychiatric disorders.^17^

The majority of longitudinal TBI outcome studies, particularly those focused on older adult populations, have been conducted in civilian populations.^18^ Compared with their civilian counterparts, TBI occurs at a greater proportion in active duty military/veteran populations.^19^ Military populations may also have an increased risk for ongoing symptoms^20^ and comorbid health conditions like posttraumatic stress disorder, depression, and problematic substance or alcohol use,^20^, ^21^, ^22^ which could increase disability or functional consequences of TBI over time. Longitudinal examination of TBI in older adult veteran populations is warranted, as these individuals may be living and aging with brain injury for longer periods of time than their predecessors.^4^ Examining the trajectories of motor and cognitive functioning after TBI among older adults—as well as their predictors—is critical for understanding the treatment and rehabilitation needs of this unique and growing population to maximize long‐term outcomes. Given this need, the aim of this study was to identify predictors of functional independence trajectories over the 5 years post‐TBI in a subset of the Veterans Affairs TBI Model Systems (VA TBIMS) study.

## METHOD

### Procedure

This study retrospectively analyzed a subset of data from the VA TBIMS study database, which examines outcomes of individuals admitted to one of the five VA polytrauma rehabilitation centers (PRCs) for inpatient rehabilitation.^23^ A different institutional review board approved VA TBIMS study design and procedures for each site.

### Participants

The VA TBIMS database inclusion criteria are (1) a diagnosis of mild, complicated mild, moderate, or severe TBI; (2) admission to a PRC with a diagnosis of TBI; (3) 16 years of age or older at injury; (4) inpatient rehabilitation and acute care in a PRC; and (5) patient or legally authorized representative gave informed consent. Data collection for the larger study began in 2009, and as of October 2021, there were 1704 participants in the database. To be included in the current study, participants must have sustained their TBI at age 55 or older and completed at least one Functional Independence Measure (FIM) Motor and Cognitive measure at the time of their 1, 2, and/or 5‐year postinjury follow‐up. Sample size was not determined prospectively and all eligible older adult participants meeting these criteria (*n* = 184) were included, with data collected from the current study sample from 2010 to 2021.

### Measures

#### Sociodemographic Characteristics

Age at injury was extracted from electronic medical records. Sex was reported as male or female. Race or ethnicity was recategorized as White versus Other due to low representation of non‐White racial identities in the dataset. Education level at time of injury was reported in years from 1 to 20. Relationship status was recategorized as partnered (married or cohabiting at the time of injury) or unpartnered. Insurance was dichotomized as private or other (ie, Medicare, Medicaid, Worker's Compensation, VA, etc.). Participants were considered employed at injury if they reported competitive employment in the month before injury. Problematic substance use was based on the participant's self‐report of a history of preinjury substance use disorder. Participants' physical and mental health prior to sustaining their TBI was categorized dichotomously based on whether they endorsed limitations in learning, dressing, working, or going out of the house. History of treatment for mental illness was self‐reported. All measures asked of participants and all available answer choices can be found in the TBIMS National Data and Statistical Center Data Dictionary.^24^

#### Injury Severity

Length of posttraumatic amnesia (PTA) was used as an indicator of injury severity. PTA has been found to be a more meaningful predictor of functional outcomes after TBI than other indicators of injury severity.^25^ Duration of PTA was either prospectively tracked during inpatient rehabilitation via a standardized measure such as the Orientation Log^26^ (*n* = 20) or Galveston Orientation and Amnesia Test^27^ (*n* = 18) or retrospectively abstracted (*n* = 90) from the participant's acute care medical record by a data collector trained on standardized TBIMS conventions (eg, consistent bedside documentation of mental status or Glasgow Coma Scale scores within a discrete period of time). Injury severity based on duration of PTA was categorized using VA/Department of Defense clinical practice guidelines^28^ as mild (0–1 days), moderate (2–7 days), or severe (8+ days). Participants with missing PTA (*n* = 56) were still included in the current study based on the approaches to handling missing data noted described subsequently. Table 1 includes the PTA values for all participants with PTA data.

**TABLE 1:** Sample characteristics.

Characteristics	(*N* = 184)
Age at injury (years), *M* (SD)	64.2 (6.8)
Sex, *n* (%)	
Male	178 (96.7)
Female	6 (3.3)
Race or Ethnicity, *n* (%)	
White	136 (73.9)
Black	20 (10.9)
Hispanic	14 (7.6)
Asian	1 (0.5)
American Indian	2 (1.1)
Multiracial/multiethnic	5 (2.7)
Education, *M* (SD)	13.4 (2.7)
Relationship status *n* (%)	
Partnered	93 (50.5)
Unpartnered	91 (49.5)
Insurance	
Private	36 (23.5)
Other (including Veterans Affairs)	117 (76.5)
Employment at injury, *n* (%)	
Competitively employed	74 (40.2)
Not employed	110 (59.8)
Active duty at injury, *n* (%)	4 (2.2)
Pre‐TBI health, *n* (%)	
Limitations to learning	26 (14.3)
Limitations to dressing	8 (4.4)
Limitations to working	31 (16.9)
Limitations to going out of the home	7 (3.8)
History of mental illness treatment	93 (50.5)
Problematic substance use	67 (37.9)
Days in PTA, *M* (SD)	43.6 (76.9)
PTA categories, *n* (%)	
Mild (0–1 days in PTA)	43 (34.1)
Moderate (2–7 days in PTA)	10 (7.9)
Severe (8+ days in PTA)	73 (57.9)
Glasgow Coma Scale scores, *n* (%)	
3	12 (9.4)
5	1 (.8)
6	3 (2.3)
7	4 (3.1)
8	3 (2.3)
9	2 (1.6)
10	3 (2.3)
11	2 (1.6)
12	3 (2.3)
13	12 (9.4)
14	36 (28.1)
15	47 (36.7)
Functional independence measure score at rehabilitation discharge, *M* (SD)	101.5 (21.4)
Cause of TBI, *n* (%)	
Motor vehicle	29 (15.8)
Motorcycle	43 (23.4)
Bicycle	11 (6.0)
All‐terrain vehicle/cycle	4 (2.2)
Gunshot wound	1 (0.5)
Assaults with blunt instrument	8 (4.3)
Other violence	7 (3.8)
Fall	66 (35.9)
Hit by falling/flying object	4 (2.2)
Pedestrian	9 (4.9)
Other	1 (0.5)
Unknown	1 (0.5)

*Note*. Not all frequencies within a demographic or injury‐related category add up to the total sample size due to missingness. Means±SDs were calculated based on available values for that variable. Abbreviations: PTA, posttraumatic amnesia; TBI, traumatic brain injury.

#### Functional Independence Measure

The FIM is a well‐established measure for assessing functional level in a number of populations,^29^ including individuals with TBI^30^ and older adults.^31^ It consists of 18 items, measured from 1 to 7 (score range: 18–126), rating an individual's level of independence in cognitive (5 items) and motor functioning (13 items). Items assessing cognition include comprehension, social interaction, problem solving, expression, and memory. Items assessing motor functioning tap eating, bathing, grooming, lower body dressing, upper body dressing, bladder and bowel management, toileting, locomotion, transfers between bed and chair and to toilet and shower, and stair climbing. Higher item and total scores indicate greater independence. The FIM was collected at each follow‐up period by a FIM‐certified data collector.

### Data Analyses

IBM SPSS Statistics version 27 was used to conduct all analyses. Little's missing completely at random (MCAR) test was performed on outcome data, and missing values were accounted for using full information maximum likelihood (FIML) estimation. This allowed all participants who met inclusion criteria to be retained, including those who had died during the follow‐up period. A reference point of 0 was given to dichotomous variables (given that the models used require continuous or dichotomous variables), and continuous variables were centered to reduce multicollinearity and make the intercept more interpretable.

Two series of hierarchical linear models (HLMs), one for each FIM subscale, were run to examine trajectories of functional independence across the 5 years after TBI in older veterans. An unconditional growth model was first run before the addition of time‐squared to examine whether linear or quadratic functional independence trends occurred over time. −2 log likelihoods (−2LLs) were compared between models, and a decrease of 3.84 chi‐squared points reflected a statistically significant (*p* < .05) improvement. The −2LL model statistics are presented in Table 2 and there was not a statistically significant improvement between the linear and quadratic models for either FIM Motor or FIM Cognitive scores.

**TABLE 2:** −2 Log likelihood values.

	FIM motor	FIM cognitive
Linear model	2576.03	1985.29
Quadratic model	2576.03	1984.69

Abbreviation: FIM, Functional Independence Measure.

Baseline predictors of the FIM Motor and FIM Cognitive trajectories among older veterans in the first 5 years after TBI were identified using HLM. After being centered or given a reference point of 0, based on whether a variable was continuous or dichotomous, baseline predictors were entered simultaneously as fixed effects with time. Predictors were considered significant if *p* < .05, and time‐squared was not included in any of the models as there was not a significant improvement using the quadratic model. Unstandardized b‐weights were generated for each predictor. Each secondary HLM examined whether outcome trajectories across the three time points could be predicted by baseline demographic and injury characteristics of: time (0 [1 year], 1 [2 years], and 4 [5 years]); age (continuous); sex (0 = female, 1 = male); race or ethnicity (0 = racial or ethnic minority, 1 = White); education level (continuous); marital status (0 = unpartnered, 1 = partnered); insurance type (0 = other, 1 = private); employment at injury (0 = not employed, 1 = employed); problematic substance use history (0 = no, 1 = yes); pre‐TBI functional limitations in learning, dressing, working, or going out of the home (0 = no limitations, 1 = limitations); history of mental health treatment (0 = no, 1 = yes); days in PTA (0 = 0–1 days, 1 = 2–7 days, 2 = 8+ days); combat experience (0 = did not see combat, 1 = saw combat); and violence (eg, gunshot, assault, or other violence) as a cause of injury (0 = nonviolent cause, 1 = violent cause). Each predictor's effect in an HLM is a unique effect, controlling for all other effects in the model. Follow‐up HLMs were conducted to test for differential effects of the predictors over time (eg, differences in FIM slope as a function of the predictor). These included only the previously significant predictors from the first primary model, time, and the interaction between time and the significant predictor. For these significant main and interaction effects, figures were created showing FIM scores over time as a function of the predictor. To avoid biasing figures by graphing outcome scores of participants with data present, missing values were imputed using expectation maximization that were then used to calculate the overall means presented in the figures.

## RESULTS

### Participant Demographics

Full demographic information can be found in Table 1. Notably, the sample consisted primarily of older White males, with ages ranging from 55 to 91 years with approximately 13 years of education. For additional context, of the participants who reported residence data at Year 1, 84.1% were living in the community and 7% were in long‐term care facilities (a number of other miscellaneous short‐term categories also were represented). At Year 2, 84.1% were living in the community and 10.9% were living in long‐term care facilities. At Year 5, 87.5% were living in the community and 12.5% were living in long‐term care facilities.

### Little's MCAR Test

Little's MCAR test was not significant, *χ* ^2^ (40) = 45.19, *p* = .264, suggesting that the FIM Motor and Cognitive data were MCAR. Nonetheless, FIML accounted for the missing FIM outcome data. At years 1, 2, and 5, the percentages of data present for FIM Motor were 72.3%, 62.0%, and 35.3% and for FIM Cognitive were 72.3%, 62.5%, and 36.4%, respectively.

### FIM Motor

**FIGURE 1:**
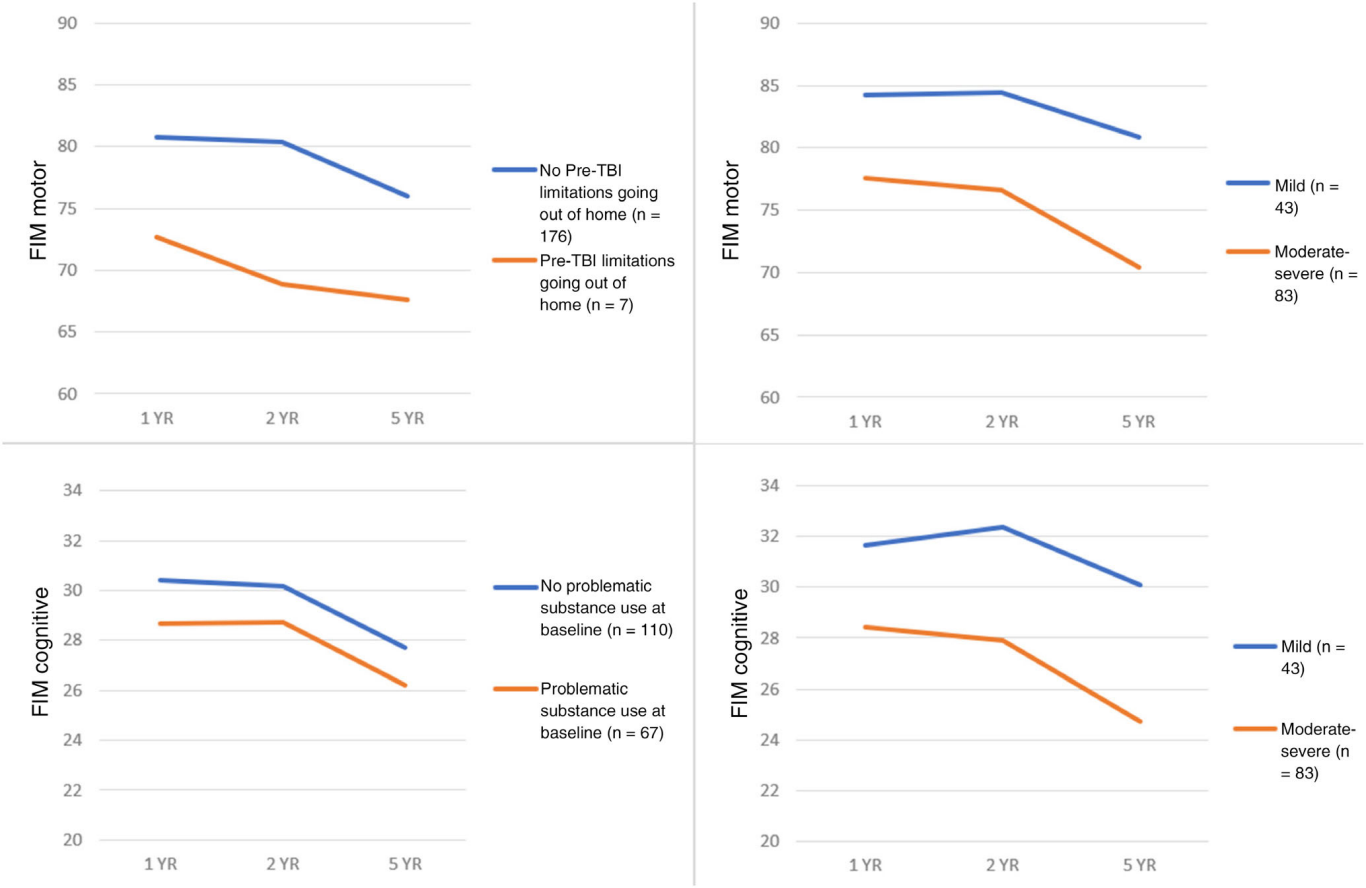
Predictor effects on FIM trajectories. FIM scores were modeled over time as a function of significant predictors. All trajectories were linear, and figures show mean scores over time using expectation maximization to impute missing FIM data to avoid biasing figures. For visualization purposes, PTA was dichotomized as mild vs. moderate and severe, although the three levels were used in the HLMs. Abbreviations: FIM, Functional Independence Measure; HLM, hierarchical linear model; PTA, posttraumatic amnesia; TBI, traumatic brain injury.

Comparison between −2LLs of models with linear and quadratic time demonstrated that FIM Motor trajectories were best characterized by linear, or straight line, movement. FIM Motor scores decreased over time (*b* = −1.66, *SE* = 0.57, *p* = .005). The *b*‐weights and *p* values for the HLM are presented in Table 3. Lower FIM Motor trajectories (ie, lower average scores collapsed across time) were seen among participants who had pre‐TBI functional limitations in going out of the home (*b* = −21.4, *SE* = 9.89, *p* = .033, Figure 1 upper left) and those with greater injury severity based on PTA (*b* = −5.17, *SE* = 1.82, *p* = .006, Figure 1 upper right).

**TABLE 3:** Fixed effects.

Characteristics	FIM motor			FIM cognitive		
	*b*‐weight	*SE*	*p* value	*b*‐weight	*SE*	*p* value
Intercept	101.38	14.26	<.001	39.51	4.72	<.001
Time	−1.66	0.57	.005	−0.82	0.23	.001
Age	−0.21	0.33	.532	−0.04	0.11	.715
Sex (1 = male, 0 = female)	−8.62	9.98	.389	−5.01	3.34	.136
Race or ethnicity	−1.04	4.33	.811	−0.38	1.42	.787
Education	0.07	0.66	.915	0.1	0.22	.652
Relationship status	0.05	3.52	.988	0.32	1.17	.786
Insurance status	−3.64	4.04	.369	−2.31	1.32	.084
Problematic substance use	−6.07	3.31	.07	−2.36	1.09	.033
Pre‐TBI health limitations						
Learning	−3.64	5.22	.488	0.78	1.71	.652
Dressing	−5.78	8.04	.475	−0.48	2.63	.854
Going out of the home	−21.4	9.89	.033	−1.17	3.26	.722
Working	3.51	5.13	.496	−0.3	1.67	.858
Mental health treatment	−1.25	3.76	.741	−1.01	1.24	.419
Posttraumatic amnesia severity	−5.17	1.82	.006	−2.39	0.6	<.001
Combat status	−0.41	3.31	.902	−0.34	1.09	.755
Employment at injury	−1.09	4.07	.79	−0.28	1.34	.833
Violent cause of injury	2.49	7.66	.745	0.56	2.47	.82

Abbreviations: FIM, Functional Independence Measure; TBI, traumatic brain injury.

Follow‐up HLMs examined whether FIM Motor linear trajectories could be predicted by the significant predictors and their time interactions (Table 4). There was a significant interaction for time * PTA (*b* = −1.91, *SE* = 0.55, *p* = .001), suggesting the relationship between PTA and motor functioning varied over time such that FIM Motor scores decreased over time among those with mild injury severity as well as for those with moderate or severe severity, but the decrease was steeper for those with a moderate or severe injury (Figure 1 upper right). There was not a significant interaction for time * pre‐TBI limitations going out of the home (*b* = 3.36, *SE* = 2.04, *p* = .101), such that the slopes of the FIM Motor decreases were similar for those with and without limitations going out of the home prior to TBIs.

**TABLE 4:** Interactions in significant predictors over time.

Interaction effects	*b*‐weight	*SE*	*p* value
FIM Motor			
Time * Pre‐TBI limitations in going out of the home	3.36	2.04	.101
Time * Posttraumatic amnesia severity	−1.91	.55	.001
FIM Cognitive			
Time * Problematic substance use	.09	.32	.783
Time * Posttraumatic amnesia severity	−.61	.23	.008

*Note*. All main effect predictors within the interaction terms were also included but not presented here for parsimony. Abbreviations: FIM, Functional Independence Measure; TBI, traumatic brain injury.

### FIM Cognitive

Comparison between the −2LLs of the models with linear and quadratic time found that FIM Cognitive trajectories were best characterized by linear, or straight line, movement. In the primary HLM, FIM Cognitive scores decreased across time (*b* = −0.82, *SE* = 0.23, *p* = .001). The *b*‐weights and *p* values are presented in Table 3. Lower FIM Cognitive trajectories (ie, lower average scores collapsed across over time) were seen among participants who had problematic substance use at baseline (*b* = −2.36, *SE* = 1.09, *p* = .033, Figure 1 lower left) and among those who had greater injury severity based on PTA (*b* = −2.39, *SE* = 0.6, *p* < .001, Figure 1 lower right).

Follow‐up HLMs investigated whether FIM Cognitive linear trajectories could be predicted by the previously significant predictors and time interactions (Table 4). There was a significant interaction for time *PTA (*b* = −.61, *SE* = 0.23, *p* = .008), reflecting differential change across time in cognitive functional independence based on injury severity. FIM Cognitive scores decreased at a steeper rate for participants with greater injury severity (Figure 1 lower right). There was not a significant interaction for time * problematic substance use at baseline (*b* = .09, *SE* = 0.32, *p* = .783).

## DISCUSSION

This study examined trajectories and correlates of longitudinal motor and cognitive functioning in the first 5 years after TBI in older veterans in the VA TBIMS study. In this sample of older veterans, motor and cognitive functioning declined over time in the years after TBI, which is consistent with data in the civilian TBI literature.^6^ The strongest predictor of functional motor and cognitive decline was duration of PTA, such that declines in functioning over time were more pronounced for those who were in PTA longer. Although preinjury limitations in leaving the home and preinjury problematic substance use were associated with lower motor and cognitive independence trajectories (lower scores over time), respectively, neither preinjury factor influenced rate of decline in these outcomes over time. Additionally, it is important to note that only seven participants endorsed preinjury limitations in leaving the home and this finding should be interpreted with caution.

Motor functioning decreased over time for all participants. Participants with longer duration of PTA showed steeper declines in FIM Motor scores, which was consistent with studies finding that functional independence is associated with injury severity^32^ and predicted by PTA.^33^ However, recent studies by Hammond and colleagues examined functional ability from 5 to 10^7^ and 5 to 15^14^ years post TBI and found that though injury severity was associated with FIM changes, it did not necessarily predict declines in functionality. It is possible that these differences in our findings may be accounted for by participant demographic characteristics (our sample was limited to veterans who had sustained a TBI at 55 or older whereas Hammond's looked at civilians across the lifespan) and/or relative stabilization of motor function at time periods further out from injury. Our findings may also be partially attributed to increased functional limitations associated with older age,^34^ as most older adults acquire one or more chronic health conditions^35^ as they age. Older adults are more likely to be homebound or have difficulty leaving the home, particularly if they are over age 75 or have one or more chronic conditions, such as a TBI.^36^ Changes in functional ability for older adults are reflected in the current findings that veterans who had difficulty leaving the home prior to TBI showed lower FIM Motor trajectories, consistent with findings from an older adult civilian study.^33^

Cognitive functioning also demonstrated a decline, consistent with previous research demonstrating declines in cognitive functioning over time^6^ in an older adult civilian population with TBI. Comparatively, research on younger adult civilians with a TBI suggests that FIM cognitive scores usually remain the same or improve over time, with only a small minority of participants experiencing a decline, regardless of injury severity.^7^ By comparison, as people age, they are likely more vulnerable to cognitive decline following TBI, especially those with more severe injuries. The current finding of both lower and steeper cognitive decline for older adult veterans with longer PTA adds to and is consistent with previous research suggesting that severity of PTA predicts lower overall FIM scores.^25^ The finding that older adult veterans with problematic substance use at the time of TBI demonstrated lower FIM Cognitive trajectories is a novel finding in the context of previous literature that has not found a link between substance use and FIM Cognitive scores when looking across age groups, suggesting that substance use at the time of injury may have more of an influence on rehabilitation trajectories for older adults in particular.^37^, ^38^, ^39^, ^40^

Clinically, these findings provide rehabilitation clinicians with a better understanding of baseline predictors of the trajectories of functional independence in older veterans with TBI. Aging veterans already have increased risk of some negative long‐term effects to physical and mental health due to military service and combat exposure without accounting for TBI.^41^ Clinicians should monitor and follow up with older veterans during this time after TBI, particularly with individuals who have experienced severe injury, have pre‐existing limitations leaving the home or substance use issues, as current findings suggest these individuals are at elevated risk of lower functional independence or even decline. Physical or cognitive rehabilitation strategies could be more aggressively deployed, even in the early phases of rehabilitation, for older veterans with these particular baseline risk factors. Given that falls were the most common mechanism of injury in the current sample, prevention efforts in older veterans are critical. Efforts to reduce incidence of TBI in this group, including the use of home‐telehealth strategies to assess home safety and fall prevention, would likely be extremely beneficial in preventing TBI before it happens,^42^ including involving caregivers.^43^

### Limitations and Future Directions

Although the current study had important findings related to post‐TBI outcomes for older veterans aging with TBI, there are several limitations to consider in interpreting and generalizing the results. Though the VA TBIMS program^44^ is one of the largest longitudinal studies of veterans and service members with TBI, the sample of adults 55 years or older was still relatively small and therefore limited in its ability to adequately capture potentially important subgroups of individuals whose trajectories may differ from the majority. The number of women was very limited (3.3%). Though this is comparable to the proportion of veterans over 75 who are women (3%), it is lower than the proportion of those between 55 and 64 (13%) and 65–74 (6%) as of 2021.^45^ Further, the proportion of women service members continues to grow substantially^45^ (18% between 18 and 34 were women as of 2021). Similarly, racial diversity was limited within our sample (73.9% White), though this is also comparable to the current racial representation within the military. Therefore, we urge caution in generalizing findings to aging women veterans and racial or ethnic minority veterans whose functional trajectories may differ from men, White veterans, or those from more recent service eras. Similar caution should be exercised when generalizing findings to all veterans, as veterans enrolled in the VA PRC database may represent a unique subset of all veterans; recent evidence indeed suggests that VA versus civilian inpatient rehabilitation systems serve distinctly different veteran/service member populations with different risk profiles. Relatedly, there was a fairly substantial loss of complete data at the 5‐year time point, with just over one third of participants having FIM scores. FIML was used to account for missing values and retain all participants, so differential attrition of the study sample is not likely to have contributed substantially to the study results. Nonetheless, a greater sample proportion of complete data might have generated more accurate parameter estimates for the moderate number of predictors in the main model.

Additionally, many of the predictor variables were self‐reported, making them subject to validity concerns and potential recall bias. The lack of baseline FIM scores in the current study for all participants and the use of PTA as the only indicator of injury severity may also limit the generalizability of results relative to more robust measures of severity; however, PTA has been shown to be the strongest injury‐severity predictor of FIM scores after TBI.^25^ Finally, our focus on predictors from time of injury provides important knowledge about prognostic factors that can inform risk stratification and planning for treatment and long‐term care. However, it does not account for effects of intervention, social and caregiving support, comorbid health conditions, or other social and environmental factors that can affect long‐term functional trajectories over time. This represents an important avenue for future research.

### Conclusion

The current study provides unique insight into the long‐term trajectories of motor and cognitive function experienced by older veterans who sustained a TBI at age 55 or older. Decline was observed over the first 5 years postinjury in both motor and cognitive function, and rate of decline was most pronounced among those with more severe TBI as characterized by PTA. These findings converge with previous studies in civilian samples to indicate that older adults with TBI are at risk for age‐related decline that warrants aggressive rehabilitation and long‐term care to optimize health and longevity. This study's findings contribute to a better understanding of post‐TBI functional rehabilitation trajectories in older adults that may inform treatment considerations to mitigate the effects of age‐related disparities in rehabilitation outcomes.

## DISCLOSURES

The authors report no conflicts of interest.
